# Emergency cervical cerclage in delayed-interval delivery of twin pregnancies: a scoping review

**DOI:** 10.1186/s12884-024-06515-x

**Published:** 2024-04-26

**Authors:** Hong Cui, Huan Li, Zhihua Yin

**Affiliations:** 1 Department of Gynaecology and Obstetrics, Shengjing Hospital Affiliated to China Medical University, Shenyang, Liaoning 110004 People’s Republic of China; 2 Department of Epidemiology, School of Public Health, China Medical University, Shenyang, Liaoning People’s Republic of China

**Keywords:** Twin pregnancy, Cervical cerclage, Delayed-interval delivery, Pregnancy outcomes

## Abstract

**Background:**

The protocol for delayed-interval delivery of the second twin in twin pregnancies has not been standardized. Cervical cerclage is often performed, but its use is debated. To conduct a scoping review on cervical cerclage for prolonging the intertwin delivery interval and improving second twin survival and maternal outcomes after preterm delivery or spontaneous abortion of the first twin in twin pregnancies.

**Methods:**

Seven Chinese and English language databases were searched from inception to March 1, 2023, including PubMed, The Cochrane Library, Web of Science, CNKI, Wanfang Data, VIP Chinese Science Journal Database, and Sinomed. Relevant observational studies that assessed the effectiveness of the use of cervical cerclage in delayed-interval delivery of twins were screened and selected, and raw data were extracted, and descriptive statistics and chi-square analysis were performed.

**Results:**

A total of 102 articles were retrieved. After screening and exclusion of duplicate and irrelevant articles, 22 articles meeting the inclusion criteria were obtained. Studies in which cerclage was performed reported longer intertwin delivery intervals than those that did not perform cerclage, and the difference was statistically significant. The cerclage group also tended to have lower rates of chorioamnionitis and maternal complications, but the difference between the two groups was not statistically significant.

**Conclusion:**

After excluding patients with contraindications, emergency cervical cerclage can be considered in cases of spontaneous abortion of the first twin in twin pregnancies to prolong the gestation and improve the prognosis of the remaining fetus until it becomes viable and increases its birth weight.

## Introduction

Delayed-interval delivery of the twin (DIDT) is a procedure in twin pregnancies in which, after spontaneous delivery or abortion of the first twin (F1) in the second trimester, fetal preservation measures are taken to keep the remaining twin (F2) in the uterus for several days or weeks until its organs are more mature before delivery. Prolonging the intertwin delivery interval (≥ 24 h) increases the chances of F2 survival [1]. In recent years, the number of multiple pregnancies has increased with the rapid development of assisted reproductive technologies and later age of women at marriage. Spontaneous preterm birth is common in multiple pregnancies [2]. Since preterm neonates usually have longer durations of hospitalization and higher risk of serious complications or even death, it is critical to take appropriate means to retain F2 in the mother as long as possible until full term. No universally accepted optimal protocol for the standardized management of delayed-interval delivery of F2 exists, and common clinical management involves preventing maternal infection, promoting fetal lung maturation, using tocolytics, and cervical cerclage.

The use of cervical cerclage after delivery of F1 to achieve delayed-interval delivery of F2 was proposed in 1956. The Shirodkar and McDonald techniques are two commonly used transvaginal cerclage procedures in clinical practice, in addition to transabdominal cerclage or laparoscopic procedures [3]. Although cervical cerclage has been in the forefront for decades, there is still debate regarding its use in delayed-interval delivery.

One view is that the use of cervical cerclage helps to close the dilated cervix after spontaneous abortion of F1, reducing the exposure of the membranes to bacteria and the acidic environment in the vagina while increasing cervical stability [4], thereby reducing the risk of premature membrane rupture and inflammation to prolong the gestational period and improve the survival and outcome of the remaining fetus. Several studies have described the successful use of cervical cerclage in delayed-interval delivery. Another view questions the safety of cervical cerclage: infection may occur during the procedure, and the operation may stimulate contractions or trigger premature membrane rupture, which is not conducive for prolonging the interval between deliveries [5, 6] and should be considered carefully depending on actual conditions.

In this study, we used the JBI scoping review [7] and the PRISMA-SCR guidelines [8] to investigate the current status and effectiveness of the use of cervical cerclage in delayed-interval delivery of twins.

## Methods

### Inclusion and exclusion criteria

The inclusion criteria were determined based on the principle of population, intervention, comparison, and outcomes. The study population was pregnant women with twin pregnancies and their neonates, the intervention was emergency cervical cerclage, the comparison was other conservative therapies including administration of antibiotics or tocolytics, promotion of fetal lung maturation, fetal neuroprotection, and/or strict bed rest instead of cervical cerclage. Outcomes included mean interval duration in days, comparison of intervals among cerclage versus non-cerclage patients, F1 and F2 mortality rates, incidence of chorioamnionitis in cerclage patients, incidence of complications in cerclage patients, neonatal intensive care unit admission rates in cerclage patients, and F2 1-minute and 5-minute Apgar scores in cerclage patients.

Excluded were: (1) comments, guidelines, websites, opinions, protocols, conference proceedings, research proposals, policy papers, and letters to the editor; (2) articles for which the full text was not available; (3) duplicate publications; (4) articles not in English or Chinese; (5) reviews or meta-analyses; (6) articles in which cervical cerclage was not used; (7) articles focused on comparing different cerclage procedures or timing; (8) articles that included data on triple or higher-order multiple pregnancies without reporting data on twin pregnancies separately; (9) articles where only prophylactic cerclage was used; and (10) articles focusing on the efficacy of cerclage in cervical insufficiency.

### Literature search

**Fig. 1:**
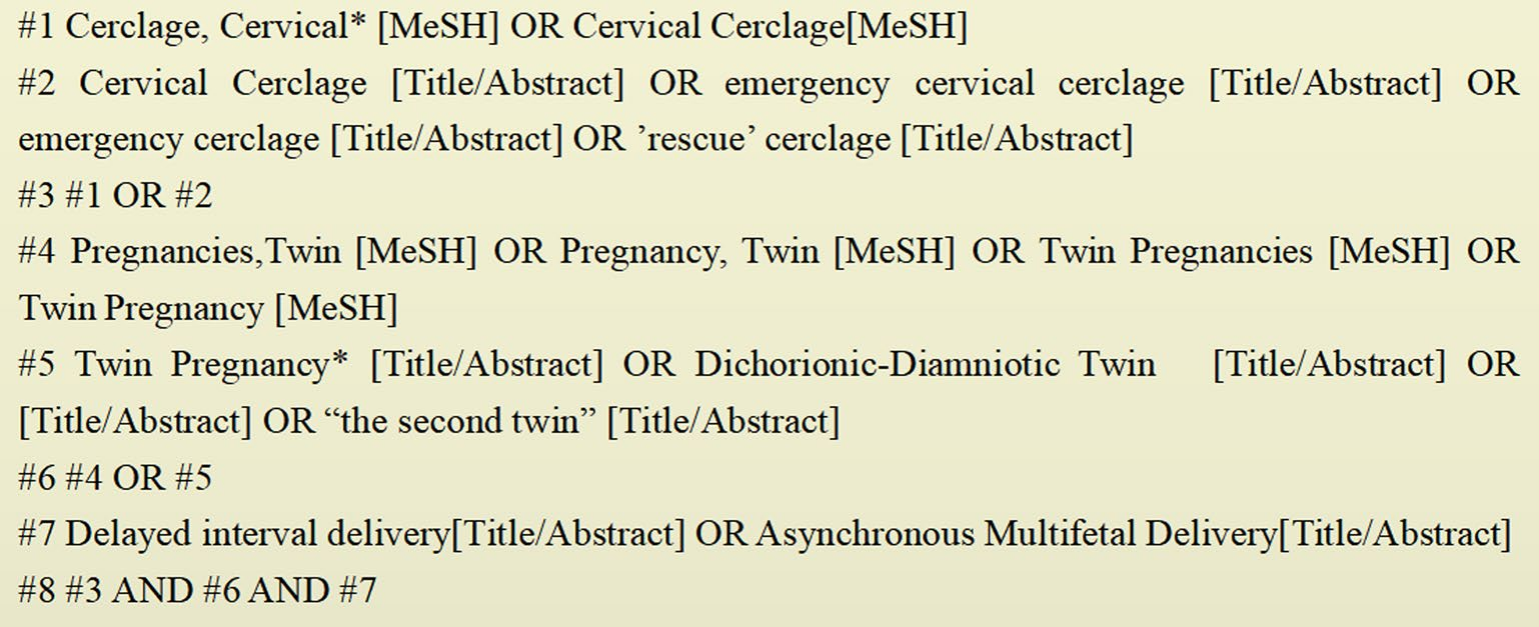
PubMed literature search strategy

The Chinese and English language literature before March 1, 2023 was searched in seven databases: PubMed, The Cochrane Library, Web of Science, CNKI, Wanfang Data, VIP Chinese Science Journal Database, and Sinomed. A combination of subject terms and free words were used for the literature searches in the English-language databases. The PubMed search strategy is shown in Fig. 1. For the Chinese-language databases, the search strategy was (SU = (“twin pregnancy” + “multiple pregnancy”) OR SU = (“delayed delivery” + “delayed-interval delivery”) OR SU=(“cervical cerclage” + ”emergency cervical cerclage”)) AND TKA=( “twin pregnancy” + “multiple pregnancy”) AND TKA=(“cervical cerclage” + “emergency cervical cerclage”) AND TKA=(“delayed delivery” + “delayed-interval delivery” + “asynchronous multiple delivery” + “asynchronous delivery”).

### Article screening and data extraction and analysis

The retrieved articles were imported into NoteExpress for de-duplication, and two researchers with experience in evidence-based practice independently conducted primary screening of the titles and abstracts of the articles based on the inclusion and exclusion criteria. Secondary screening was performed by reading the full text of qualified articles. The researchers independently extracted author and publication date, country, study type, sample size, and outcome indicators. Disagreements arising during screening and data extraction were discussed and resolved by a third investigator.

## Results

### Literature search results

**Fig. 2:**
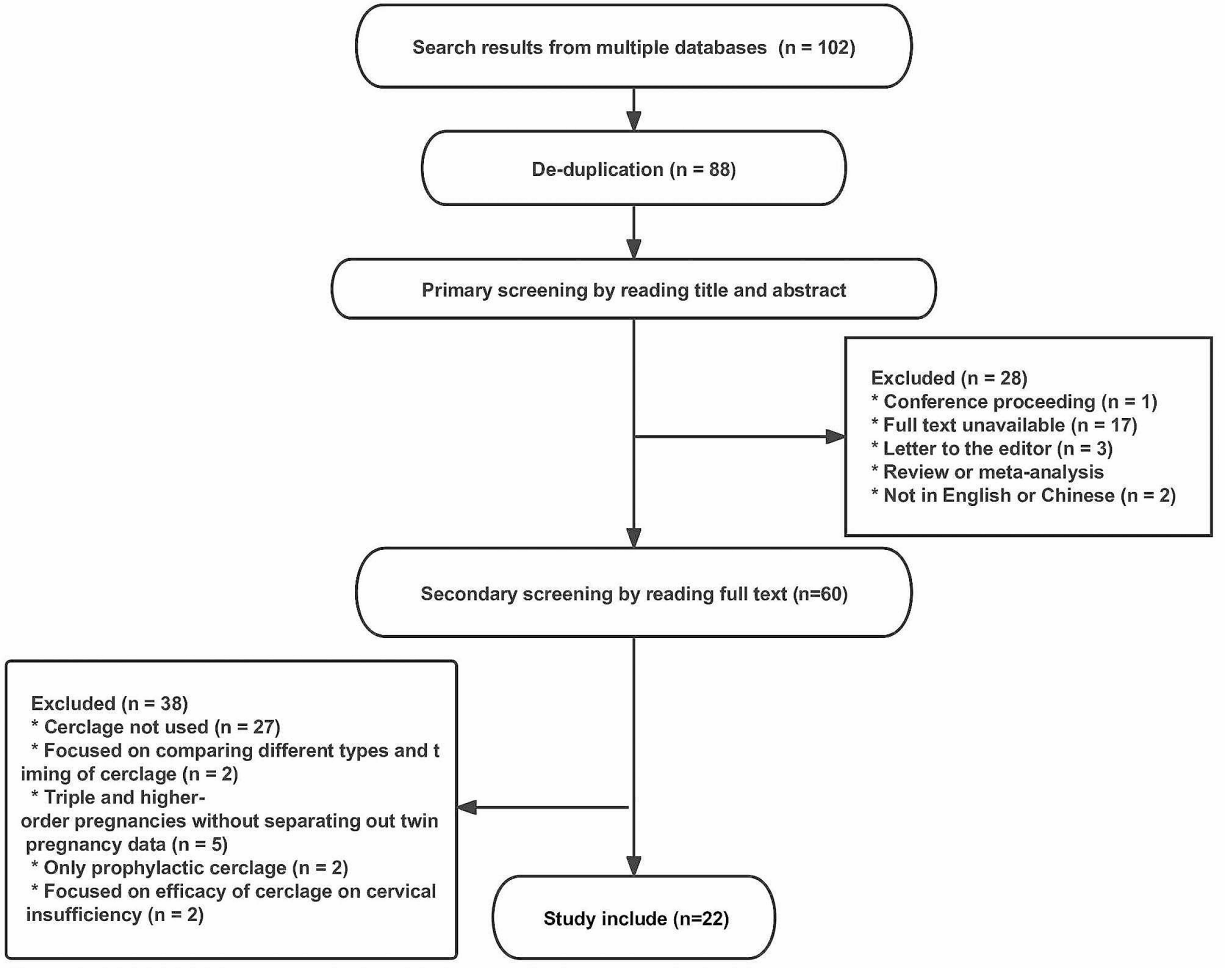
Flowchart of article screening

A total of 102 papers were retrieved. The following articles were excluded: 14 duplicates; 28 that failed primary screening, including 1 conference proceeding; 17 for which the full text was unavailable, 3 letters to the editor, 2 not in English or Chinese, and 5 reviews and meta-analyses. After reading the full text, 38 papers were removed, including 27 articles in which cerclage was not actually applied but were only mentioned when citing other studies, 2 articles that focused on comparing different types (prophylactic/emergency/McDonald/Shirodkar) and timing of cerclage, 5 articles that recorded data on triple or higher-order multiple pregnancies without considering twin pregnancies separately, 2 articles that used only prophylactic cerclage, and 2 articles that focused on describing the efficacy of cerclage on cervical insufficiency. Literature on the efficacy of cervical insufficiency was excluded. Finally, 22 articles were included. Figure 2 shows a flowchart of the screening process.

### General characteristics of included studies

Among the 22 articles included in the study, 15 were case reports and 7 were case series analyses. Six reports were from China (including one case from Taiwan, China), three from France, three from Turkey, two from India, two from Greece, and one each from Portugal, the United Kingdom, South Korea, Japan, Cameroon, and Nigeria. In total, 82 women with delayed-interval delivery in twin pregnancies, of which 43 underwent emergency cerclage and 39 underwent only conservative treatment were reported. Delayed-interval delivery was generally considered to be more appropriate for dichorionic diamniotic twins. Some researchers suggested that in monochorionic diamniotic (MCDA) twins, F2 may suffer severe neurological damage after delivery of F1 due to reduced placental perfusion or chorioamnionitis via communicating placental vessels [9]; however, some successful cases of delayed-interval delivery in MCDA twins were reported [10]. The McDonald cerclage method was used in all studies. The general information on the authors, year, country, type of study, sample size, chronicity, the type of cerclage used, and the number of cerclage and non-cerclage cases is summarized in Table 1.

**Table 1:** Characteristics of included reports (n = 82)

Included study	Publication date	Country	Type of study	Sample size (number of twin pregnancies)	Chorionicity	Type of cerclage used	Cerclage/no cerclage
Ugoj et al. [11]	2023	Nigeria	Case report	1	DCDA^*^	McDonald	1/0
Sharma et al. [12]	2022	India	Case report	1	DCDA	McDonald	1/0
Park et al. [13]	2022	South Korea	Case report	2	DCDA	McDonald	2/0
Cheng et al. [14]	2021	China	Case series	7	DCDA	——	4/3
Liu et al. [15]	2021	China	Case report	4	DCDA	——	4/0
Ngalame et al. [16]	2020	Cameroon	Case report	1	DCDA	McDonald	1/0
Cheng et al. [17]	2020	Taiwan, China	Case series	5	4 DCDA & 1 MCDA^*^	——	1/4
de Frias et al. [18]	2020	Portugal	Case report	1	DCDA	McDonald	1/0
Yu et al. [19]	2019	China	Case series	8	DCDA	——	2/6
Imachi et al. [20]	2019	Japan	Case report	1	DCDA	McDonald	1/0
Api et al. [21]	2014	Turkey	Case report	1	DCDA	McDonald	1/0
Singh et al. [22]	2012	India	Case report	1	DCDA	McDonald	1/0
Aydin, Y et al. [23]	2012	Turkey	Case report	1	DCDA	McDonald	1/0
Ding et al. [24]	2012	China	Case series	4	DCDA	——	1/3
Petousis et al. [25]	2012	Greece	Case series	5	DCDA	McDonald	5/0
Caliskan et al. [26]	2011	Turkey	Case report	1	DCDA	McDonald	1/0
Chen et al. [27]	2011	China	Case report	2	DCDA	——	1/1
Khan et al. [28]	2008	UK	Case report	1	DCDA	McDonald	1/0
Klearhou et al. [29]	2007	Greece	Case report	1	DCDA	McDonald	1/0
Cristinelli et al. [30]	2005	France	Case series	4	DCDA	——	2/2
Fayad et al. [31]	2002	France	Case series	28	——	——	9/19
Abboud et al. [32]	1997	France	Case report	2	DCDA	McDonald	1/1

^*^ DCDA: dichorionic diamniotic twin pregnancy; MCDA: monochorionic diamniotic twin pregnancy

### Outcome indicators of cervical cerclage

Table 2 summarizes the clinical outcomes of the study population.

**Table 2:** Outcome indicators in included studies (n = 82)

Included study	F1 mortality rate (%)	F2 mortality rate (%)	d^*^	d1/d2^#^	Obstetric management measures other than cerclage	Incidence of chorioamnionitis in cerclage patients (%)	Complication rate in cerclage patients (%)	F2 NICU admission rate in cerclage patients (%)	F2 1 min/5 min Apgar scores in cerclage patients (%)
Ugoj et al. [11]	100%	0%	33	33/-	Antibiotics, bed rest, tocolytics, progestogens to protect fetus	0%	0%	0%	9/10
Sharma et al. [12]	100%	0%	59	59/-	High umbilical cord ligation, antibiotics, tocolytics, progestogens to protect fetus, promoting fetal maturation, bed rest	0%	0%	100%	——
Park et al. [13]	100%	0%	86 (46, 126)	86/-	High umbilical cord ligation, tocolytics, broad-spectrum antibiotics	50%	0%	0%	7/9, 9/10
Cheng et al. [14]	100%	28.6%	79.6 ± 20.1	98.3/54.7	Antibiotics, tocolytics, progestogens to protect fetus, promoting fetal maturation, fetal neuroprotection, amnioinfusion	75%	25%	——	——
Liu et al. [15]	100%	25%	34.8 ± 12.8	34.8/-	High umbilical cord ligation, tocolytics, promoting fetal maturation, fetal neuroprotection	100%	0%	25%	8/9, 9/10, 8/8, 0/0
Ngalame et al. [16]	100%	0%	100	100/-	Progestogens to protect fetus, promoting fetal maturation, tocolytics, antibiotics, bed rest, anticoagulants	0%	0%	100%	8/-
Cheng et al. [17]	60%	0%	15.0 ± 4.8	14.0/15.3	High umbilical cord ligation, tocolytics, antibiotics, promoting fetal maturation	100%	100%	——	——
de Frias et al. [18]	100%	0%	154	154/-	Antibiotics, tocolytics	0%	0%	——	——
Yu et al. [19]	26.9%	62.5%	11.9 (3, 50)	31/-	High umbilical cord ligation, antibiotics, tocolytics, transfusion	0%	0%	——	0/0, 3/6
Imachi et al. [20]	100%	0%	83	83/-	Antibiotics, tocolytics, promoting fetal maturation	0%	0%	——	9/9
Api et al. [21]	100%	100%	4	4/-	High umbilical cord ligation, progesterone to protect fetus, promoting fetal maturation, antibiotics, tocolytics, anticoagulants	0%	0%	100%	——
Singh et al. [22]	100%	0%	104	104/-	High umbilical cord ligation, tocolytics, promoting fetal maturation, antibiotics, progestogens to protect fetus	0%	0%	0%	8/10
Aydin et al. [23]	100%	0%	101	101/-	Antibiotics, tocolytics, promoting fetal maturation	0%	0%	——	9/-
Ding et al. [24]	100%	0%	24.3 ± 7.6	7/30	High umbilical cord ligation, antibiotics, tocolytics, promoting fetal maturation	——	0%	——	——
Petousis et al. [25]	100%	20%	61.4 ± 24.6	61.4/-	Antibiotics, tocolytics	0%	0%	75%	8/9, 0/0, 6/7, 4/6, 1/4
Caliskan et al. [26]	100%	0%	72	72/0	Antibiotics, tocolytics, progestogens to protect fetus, promoting fetal maturation	0%	0%	0%	8/10
Chen et al. [27]	100%	100%	22.5 (5, 40)	5/40	High umbilical cord ligation, antibiotics, tocolytics	100%	0%	——	0/0
Khan et al. [28]	100%	0%	106	106/-	Antibiotics, promoting fetal maturation, transfusion	——	100%	0%	——
Klearhou et al. [29]	100%	0%	48	48/-	Antibiotics, tocolytics, promoting fetal maturation, bed rest, anticoagulants	0%	0%	——	4/7
Cristinelli et al. [30]	100%	25%	7 (2, 93)	50.5/4	Antibiotics, promoting fetal maturation, tocolytics, high umbilical cord ligation	50%	50%	——	-, 6/8, 3/-, 5/6
Fayad et al. [31]	92.6%	21.4%	47 ± 35.7	61.7/39.7	High umbilical cord ligation, antibiotics, tocolytics	——	——	——	——
Abboud et al. [32]	100%	50%	37.5 (8, 67)	37.5/8	Antibiotics, progestogens to protect fetus, bed rest, tocolytics	——	——	——	6/-

* d, mean duration of interval between births. SPSS 26.0 software was used for descriptive statistics of the raw data for each sample. Data conforming to the normal distribution are shown as mean ± standard deviation; data not conforming to the normal distribution is shown as median (range); # d1/d2, mean duration of interval for cerclage/non-cerclage patients. NICU, neonatal intensive care unit

### Interval duration

The duration of the interval between deliveries ranged from 3 to 154 days, with a mean of 62.7 days for cerclage patients and 20.2 days for non-cerclage patients (excluding Fayad et al. [31], see Sect. 3.3.3). Emergency cerclage patients tended to have longer intervals than non-cerclage patients; the difference was statistically significant (*p* < 0.001, Table 3). The interval in the non-cerclage patients was longer than that in the cerclage patients in only 3 reports [17, 24, 27]. Zheng et al. [17] reported the F1 placenta was not delivered in all cases, the F2 membranes were not ruptured, high ligature of the umbilical cord was performed, and antibiotics, tocolytics, and fetal lung maturation-promoting drugs were administered regardless of whether cerclage was performed. The difference was that the median gestational age of F1 at delivery was 24.7 weeks in the cerclage group and 20.9 weeks in the non-cerclage group, and the C-reactive protein (CRP) and white blood cell count (WBC) were higher in the cerclage group. In Ding et al. [24], the F1 placenta was not delivered and the F2 membranes were not ruptured in all cases, high ligature of the umbilical cord was performed, and antibiotics, tocolytics, and fetal lung maturation-promoting drugs were administered regardless of whether cerclage was performed. The mean maternal age was 35 and 29.3 years in the cerclage and non-cerclage groups, respectively, CRP and WBC were significantly higher in the cerclage group, and *Enterococcus faecalis* infection was observed in the cerclage group but not in the non-cerclage group. Chen et al. [27] reported cerclage and non-cerclage patients who both had twin pregnancies resulting from assisted reproductive technologies, and both patients underwent antibiotic therapy and fetal preservation but developed chorioamnionitis. The cerclage patient was 41 years old and had an F1 gestational age of 22.6 weeks; the non-cerclage patient was 31 years old and had an F1 gestational age of 17.4 weeks.

**Table 3:** Interval duration between the cerclage and non-cerclage groups (n = 54)

Group	Mean ± SD	Difference and 95% CI	*t*-test	
			*t*	*p*
Cerclage	62.7 ± 10.4	42.5 (21.9–63.1)	4.15	< 0.001
Non-cerclage	20.2 ± 6.7			

CI, confidence interval; SD, standard deviation

Among cerclage patients, the mean interval duration was shorter than 50 days in 9 and longer than 50 days in 13 studies. In the 9 articles reporting a shorter mean gestational interval in the cerclage patients [11, 15, 17, 19, 21, 24, 27, 29, 32], there were 13 cases of emergency cerclage, resulting in F2 death in 3 of 13 cases and F2 survival with chorioamnionitis in 6 of 10 cases (60%). The mean gestational age of F1 was 22.8 weeks and the mean maternal age was 35 years in these 9 reports. In the remaining 13 articles, there were 30 cases of emergency cerclage, resulting in F2 death in 9 of 30 cases and F2 survival with chorioamnionitis in 4 of 21 cases (19%). The mean gestational age of F1 was 22.1 weeks and the mean maternal age was 33.7 years.

### Obstetric management measures other than cerclage

Antibiotics were used in all cases in the 22 included articles. Tocolytics were used in 21 articles, fetal lung maturation-promoting drugs in 14 articles, high ligature of the umbilical cord after expulsion of F1 in 13 articles, fetal preservation with progestogens in 8 articles, bed rest in 5 articles, anticoagulants in 3 articles, magnesium sulfate for fetal brain neuroprotection in 2 articles, maternal blood transfusion in 2 articles, and amnioinfusion for oligohydramnios in 1 article.

The antibiotics used in the included studies included amoxicillin-clavulanic acid [11, 16, 24, 28, 32], cephalosporins [12, 13, 15, 17, 22], metronidazole [11–13, 26, 29], levofloxacin [17], erythromycin [25, 32], sulbactam-ampicillin [21, 25, 26], ampicillin [18], gentamicin [16, 18], non-steroidal anti-inflammatory drugs [32], and azithromycin (for mycoplasma infections) [14, 17, 24]. The tocolytics used included drotaverine [11], ritodrine [13–15, 17, 24, 25], magnesium sulfate [14, 20–25], atosiban [12, 15], nifedipine [15, 16, 21, 23], indomethacin [18, 22, 23, 26], and isoxsuprine [22]. Fetal lung maturation was promoted using dexamethasone [14, 17, 24, 28] and betamethasone [12, 15, 16, 20–23, 26, 29]. Prophylactic anticoagulation was achieved with enoxaparin [21, 29] and aspirin [16].

### Other outcome indicators

Fayad et al. [31] did not report data on the outcomes of individual cases; thus, these cases were not counted. In the remaining articles, 11 of the 34 women who underwent emergency cerclage developed chorioamnionitis (32.4%) and 5 developed maternal complications (14.7%), namely puerperal infection [14], intrauterine infection [15], postpartum hemorrhage [17, 28], and psychological disorders [30]. The 1- and 5-minute Apgar scores of F2 were taken in 25 cases that underwent emergency cerclage. In 13 of them, the 1-minute Apgar score was less than 7, indicating some degree of asphyxia. Eight of the 20 patients (40%) who did not undergo emergency cerclage developed chorioamnionitis, and 5 (25%) developed maternal complications, including sepsis [14], postpartum hemorrhage [14, 17], placental abruption [17], and placental accretion [17]. Apgar scores were not reported in most cases. The incidence of chorioamnionitis was 32.4% in the cerclage group and 40% in the non-cerclage group (*χ*^*2*^ = 0.323, *p* = 0.570). The incidence of maternal complications was 15.6% in the cerclage group and 25% in the non-cerclage group (*χ*^*2*^ = 0.884, *p* = 0.341). The differences in the risks of chorioamnionitis and maternal complications between the two groups were statistically significant (Tables 4 and 5).

**Table 4:** Cases of chorioamnionitis between the cerclage and non-cerclage groups (n = 54)

Group	Total cases	Disease [proportion (%)]	Effective [proportion (%)]	Chi-square test	
				χ^2^	*p*
Cerclage	34	11 (32.4%)	23 (67.6%)	0.323	0.570
Non-cerclage	20	8 (40%)	12 (60%)		

**Table 5:** Obstetric complications between the cerclage and non-cerclage groups (n = 52)

Group	Total (cases)	Disease [proportion (%)]	Effective [proportion (%)]	Chi-square test	
				χ^2^	*p*
Cerclage	34	5 (14.7%)	29 (85.3%)	0.884	0.341
Non-cerclage	20	5 (25%)	15 (75%)		

## Discussion

A literature search retrieved 102 potentially relevant articles. After screening and removing duplicate and irrelevant articles, 22 studies meeting the inclusion criteria remained. Among these, the studies involving cerclage procedures reported significantly longer intervals between intertwin deliveries compared to studies without cerclage. While the cerclage group also exhibited tendencies for lower rates of chorioamnionitis and maternal complications, these differences were not statistically significant compared to the non-cerclage group.

In 19 of the 22 articles, the interval duration was longer in the cerclage group than the non-cerclage group, with statistical significance. When the opposite result was obtained (3 articles), there were some cases of lower F1 gestational age and some cases of lower CRP and WBC, important predictors of chorioamnionitis, in the non-cerclage group. The cases of emergency cerclage were divided into two groups, those with a mean interval duration shorter than 50 days and those longer than 50 days. We found that the group with the longer interval duration had a lower F1 gestational age at delivery, lower maternal age, and lower incidence of chorioamnionitis than the group with the shorter interval duration given the same obstetric management measures. Thus, we hypothesize that in younger women, cases in which F1 is delivered earlier, cases without infection, and cases with emergency cerclage are more likely to have a longer interval duration and a better outcome for the remaining fetus. Although the present study has a small sample size, some previous studies may provide some support for this hypothesis. Zhan et al. [33] suggested that the gestational age of F1 largely determines the outcome of F2; the lower the gestational age of F1 at delivery and the longer the interval between F1 and F2, the better the outcome of F2. Rosbergen et al. [34] suggested that a lower gestational age of F1 is associated with a longer interval and better birth outcome of F2. Farkouh et al. [9] reported a longer delivery interval in cases of lower gestational age of F1. Similarly, de Jong et al. [35] reported better F2 outcomes in cases of early F1 delivery. de Frias et al. [18] reported the longest interval (154 days) and the lowest gestational age at delivery (15.1 weeks). Imachi et al. [20] believed that prevention of chorioamnionitis is the most important aspect of delayed-interval delivery, whereas Abboud et al. [32] suggested that infection is the factor that is the most determinative of outcomes.

Conservative management of delayed-interval delivery includes high ligature of the F1 umbilical cord with absorbable suture, antibiotics to prevent infection, tocolytics, promotion of fetal lung maturation, fetal preservation with progestogens, fetal neuroprotection with magnesium sulfate, bed rest, prophylactic anticoagulation, and so on. Singh et al. [22] and Api et al. [21] both suggested the use of absorbable suture and F1 umbilical cord ligature as close to the cervix as possible under aseptic conditions to prevent ascending infection. Rupture of the F1 amniotic sac after F1 delivery exposes the mother and F2 to the risk of ascending infection [36], and the lack of blood supply in the reproductive tract or necrotic tissue may provide opportunities for growth of *Escherichia coli*, *Streptococcus*, and *Enterococcus faecalis* and intrauterine infection [37]. Thus, most researchers recommend the selection of an appropriate antibiotic based on the results of cervical secretion culture as well as prophylactic antibiotic treatment even without indications of infection [38]. Although no standardized dosing regimen has been established, intravenous dosing for 3 days followed by oral dosing for at least one week is most common. Subsequent signs of infection, such as uterine tenderness, elevated WBP, CRP, and erythrocyte sedimentation rate, and fever, may require reintroduction of antibiotics. The success of delayed-interval delivery using emergency cerclage depends mainly on the prevention of possible subclinical infections and tocolysis [26]. Currently, tocolytics and antibiotics are both conservative treatments in the routine management delayed-interval delivery [39]. A systematic review and network meta-analysis of 95 randomized controlled trials on tocolytic therapy for preterm delivery by Haas et al. [40]. showed that calcium channel blockers (nifedipine) and prostaglandin inhibitors (indomethacin) were the most effective for prolonging the interval and improving neonatal outcomes. Calcium channel blockers were most beneficial for neonatal outcome while prostaglandin inhibitors caused the fewest maternal side effects. Magnesium sulfate and β-agonists were slightly less effective than these two, with β-agonists causing the most maternal side effects. Few cases of combined use of tocolytics have been reported, and co-administration may increase the probability of side effects. When preterm delivery risk of F2 is high, glucocorticoids can be administered to prevent the development of hyaline membrane disease and infant respiratory distress syndrome. When the fetus is less than 22 weeks of gestation with few primitive alveoli, glucocorticoids should not be used to avoid adverse effects. At a gestational age of 22–23 weeks, a course of prenatal treatment can be given. Raposo et al. [41] and Graham et al. [42] recommended promoting fetal lung maturation at F2 gestational age of 24 weeks, while Doger et al. [43] suggested that glucocorticoids should be used at F2 gestational age of 26 weeks. Louchet et al. [44] suggested that fetal lung maturation treatment should be administered from 24 weeks of gestation, and they also suggested an additional course of treatment around 28 weeks of gestation. Magnesium sulfate can protect the fetal brain and nerves and reduce the risk of cerebral palsy by stabilizing fetal cerebral circulation, and the use of magnesium sulfate for fetal neuroprotection has been reported since 1995 [45].

Bed rest is considered an alternative to cervical cerclage. Raposo et al. [41], Cozzolino et al. [46], and Doger et al. [43] concluded that strict bed rest should be applied until F2delivery; however, this is difficult to achieve and has low patient compliance, and it may cause complications such as thrombosis [28]. Five articles included bed rest, and two also included anticoagulation treatment for the mother. The American College of Obstetricians and Gynecologists Practice Bulletin No. 144 states that routine hospitalization and bed rest are not recommended for women with uncomplicated twin pregnancies and that prolonged bed rest may lead to thrombosis and deconditioning [47]. One study showed that emergency cerclage was superior to bed rest with respect to prolonging the delivery interval, preventing preterm delivery before 34 weeks and reducing the risk of neonatal complications [48].

The included articles had longer delivery intervals (but the difference was not statistically significant) and a lower incidence of chorioamnionitis among the cerclage group compared to the non-cerclage group. In a retrospective analysis of cases of delayed delivery of the retained fetus after loss of the first fetus by Doger et al. [43], 20 patients were divided into a cerclage and a non-cerclage group, and it was found that emergency cervical cerclage after delivery of F1 was associated with longer delivery intervals and higher F2 weight. Moreover, there was no statistical difference between the two groups in terms of F2 delivery week, live birth rate, take-home baby rate, and chorioamnionitis ratio. Zhang et al. [49] reviewed 66 primary reports from 7 case series and found that patients who underwent emergency cervical cerclage had statistically significantly longer delivery intervals, and emergency cervical cerclage did not significantly increase the risk of intrauterine infection after controlling for factors such as F1 gestational age, class of antibiotic, and use of tocolytics. Most researchers believe that the decision to perform emergency cervical cerclage should be made within 2 h after delivery of F1 [21, 50, 51]. Doger et al. [43]. suggested a McDonald cerclage should be placed if the cervix is effaced more than 70% or if the F2 amniotic membrane is prolapsed and needs to be pushed back into the uterine cavity, and a Shirodkar cerclage should be placed if the cervix is effaced 60% or less and the F2 amniotic membrane is not compressing the cervix. In addition, a meta-analysis of the conditions under which emergency cervical cerclage is indicated suggested that emergency cervical cerclage can help prolong pregnancy and reduce preterm delivery in cases of a cervical length of less than 15 mm in twin pregnancy or a cervical dilation of over 10 mm, whereas the benefit of emergency cervical cerclage in twin pregnancies with normal cervical length remains to be proven [52].

The most common maternal complication in delayed-interval delivery is infection, with approximately 22% of patients developing inflammatory conditions such as chorioamnionitis, thrombophlebitis, and endometritis, nearly 10% developing postpartum hemorrhage, and 6% developing placental abruption [53]. Although cervical cerclage has been suggested to increase the risk of infection and premature rupture of membranes, the included articles did not report this tendency, and patients who underwent cerclage appeared to have a longer interval between deliveries. Emergency cervical cerclage was not effective in delaying pregnancy in some of the cases analyzed here, where the mother either delivered the first fetus at a later gestational age or exhibited signs of infection. Therefore, strict control of infection is necessary to achieve the desired outcome of the cerclage.

The limitations of the present study include: (1) small sample size; (2) recall bias and measurement bias inherent to retrospective studies; (3) publication bias because articles concluding that emergency cerclage is effective are more likely to be published.

## Conclusions

Emergency cervical cerclage has been a controversial measure in obstetric management, and it is difficult to draw conclusions in the absence of prospective randomized controlled trials [35]. Herein, we reviewed 22 case reports and case series of emergency cervical cerclage in delayed-interval delivery of twin pregnancies and found that patients undergoing cerclage had longer delivery intervals than those who did not undergo cerclage; the difference was statistically significant. Patients undergoing cerclage had lower rates of chorioamnionitis and maternal complications than patients who did not undergo cerclage, but the difference between the two groups was not statistically significant. Given the experience from previous studies, we conclude that emergency cervical cerclage can be considered for delayed-interval delivery after clear communication of the risks if the mother has a strong desire to preserve the pregnancy and does not exhibit contraindications to delayed-interval delivery. A comprehensive and holistic assessment of maternal physical parameters should be performed before the procedure, and it should be performed at an appropriate time and combined with management measures such as antibiotics, tocolytics, promotion of fetal lung maturation, fetal preservation with progestogens, fetal neuroprotection, and prophylactic anticoagulation, while always keeping the patient under close observation and testing all parameters to detect clinical and biochemical evidence of infection in a timely manner.

## Data Availability

The datasets used and/or analysed during the current study available from the corresponding author on reasonable request.

## Abbreviations

MCDA: monochorionic monoamniotic
DIDT: Delayed-interval delivery of the twin
CRP: C-reactive protein
WBC: white blood cell count
